# Musicians and researchers: two creative professions striving to improve heart health through music

**DOI:** 10.3389/fcvm.2024.1536829

**Published:** 2025-01-10

**Authors:** Andrea Pozzati, Ralf Weiskirchen

**Affiliations:** ^1^ UOS Bazzano, Maggiore Cardiology Unit, Bologna, Italy; ^2^ Institute of Molecular Pathobiochemistry, Experimental Gene Therapy and Clinical Chemistry (IFMPEGKC), RWTH University Hospital Aachen, Aachen, Germany

**Keywords:** music, blood pressure, heart-brain connection, emotion, artificial intelligence

## Abstract

Musicians and researchers are creative professions that share many similarities. They both aim to bring joy and progress to humanity. In recent decades, it has been shown that music has the ability to alleviate pain, improve heart function, reduce anxiety, and stimulate the release of endogenous opioids in the brain. This has led to the emergence of music therapy as a popular therapeutic option for supporting and regulating cardiovascular health, emotional, cognitive functions, and mental wellbeing. Similarly, translational researchers and clinicians strive to generate new medical knowledge and effective treatments for various diseases. In their daily work, both musicians and scientists engage in the development of new ideas, concepts, and visions. They explore and experiment to find the best way to create something novel. Furthermore, driven by discovery, curiosity, and a deep longing, they strive to make a significant impact on heart health. In today's world, artificial intelligence technology plays an increasingly important role in achieving these goals in both fields. Success is measured by publications in esteemed journals or achieving high rankings on music charts. This review explores the similarities between researchers and musicians and how music therapy can aid in the treatment of specific symptoms related to heart function.

## 1 Introduction

Music has been one of the most important recreational activities for humans for centuries, and perhaps even for millennia. It possesses a unique characteristic of having a universal language. The sound and harmonic elements of music can be utilized to enhance the activities of individuals or groups, ranging from ceremonial situations in prehistoric times to pure entertainment in our modern era. The objectives of music are to influence the physical, emotional, intellectual, and social well-being of individuals. However, can we assert that listening to music can have an impact on the heart?

The intersection of music and medicine has a long history. In ancient Greece, Pythagoras and Plato believed that music had the power to heal both the soul and the body. During medieval times, music was employed as a treatment for various ailments, including depression, anxiety, and anguish (1, 2).

In recent decades, scientific research has started to validate the positive effect of music on health. Studies have demonstrated that music can reduce stress, enhance mood, increase motivation and concentration, and even aid in pain reduction.

A study conducted by the University of Oxford and presented at the British Cardiovascular Society in Manchester in 2015 revealed that classical music can have positive effects on hearth health. The study found that listening to famous compositions such as Va Pensiero by Giuseppe Verdi, Nessun Dorma by Giacomo Puccini, and Beethoven's Ninth Symphony can reduce heart rate and improve blood pressure (3, 4). These songs contain delicate rhythms that resemble the natural patterns that regulate the blood pressure, resulting in a calming effect. As a result, heart rate decreases and blood pressure is slightly lowered.

Cardiologists reached this conclusion by examining previous studies and analyzing the effects produced by this type of music on a group of students. In particular they examined six musical genres, and discovered that classical music with a specific rhythm of 10 s had the greatest impact in reducing blood pressure. However, they found that classical music pieces with a faster pace, such as an excerpt from Vivaldi's Four Seasons, did not have an effect on the heart and cardiovascular system (4).

In this regard, Peter Sleight, the author of the study and a cardiologist at the University of Oxford, made a statement at the British Cardiovascular Society conference: “*Music is already used as a relaxation therapy, but this work reviewed the studies on the topic and checked their effectiveness. Our research has provided improved understanding as to how music, particularly certain rhythms, can affect your heart and blood vessels. But further robust studies are needed, which could reduce skepticism of the real therapeutic role of music*”.

Since that groundbreaking statement, it has become evident that music has a significant positive impact on various health conditions, especially in improving symptoms related to heart disease and other ailments. Listening to music can lower blood pressure, decrease stress levels, and improve overall cardiovascular function by promoting relaxation and emotional well-being. Furthermore, music therapy has been used as a supportive treatment for patients with chronic illnesses, helping to reduce anxiety, pain, and depression. But what evidence supports the idea that music interventions enhance the health of individuals living with heart disease? In this article, we will briefly outline some findings and discuss examples that confirm the therapeutic effectiveness of music therapy, with a major focus on its impact on heart symptoms.

When searching for “Music and Heart” a total of 1,911 studies were identified. A search using the search terms “Music and heart and treatment” resulted in 1,126 hits (searches conducted on October 31, 2024). Similarly, a search for “Music and heart and therapy” yielded 1,088 entries (all searches conducted on October 31, 2024). The findings from the PubMed database indicate a significant body of research exploring the therapeutic potential of music and a substantial interest related to music's impact on heart treatment and therapy. This suggests that music may play a valuable role not only in broader therapeutic practices but also specifically in cardiovascular care, warranting exploration into its efficacy and applications.

## 2 Therapeutic benefits of Mozart's music

In 1993, the two neurobiologists, Frances H. Rauscher, Gordon L. Shaw, along with Catherine N. Ky, published a study that examined the short term effects of Mozart's music on a cohort of students. This cohort demonstrated an enhancement in cognitive performance after exposure to Mozart's Sonata for Two Pianos, K 448, for 10 min before completing standard IQ spatial reasoning tasks (6, 7). Another example of the benefits of music in clinical setting is the story of “Little Krissy”, a child born prematurely with significant psychomotor retardation. After attending a Mozart concert and receiving training from a skilled violin teacher, Krissy's progress was so impressive that she eventually became a violinist herself. This second example further supports the concept of the “Mozart effect” (6, 8).

Another recent study explored the impact of brief music listening on the visuospatial component of working memory (WM) in 311 young and older adults (9). Following exposure to musical excerpts from Mozart, Vivaldi, and Glass, participants' visuospatial WM was assessed using tasks like the Corsi blocks task-backwards and the Visual Patterns Test, with a silence condition included for comparison. Results showed that only Mozart's music significantly improved visuospatial WM across both age groups, particularly in the Visual Patterns Test, helping counter age-related decline in older adults (9). The findings suggest that Mozart's music possesses unique qualities that differentiate it from other composers, contributing to its distinct positive effects on visuospatial working memory in individuals, regardless of age.

**Figure 1:**
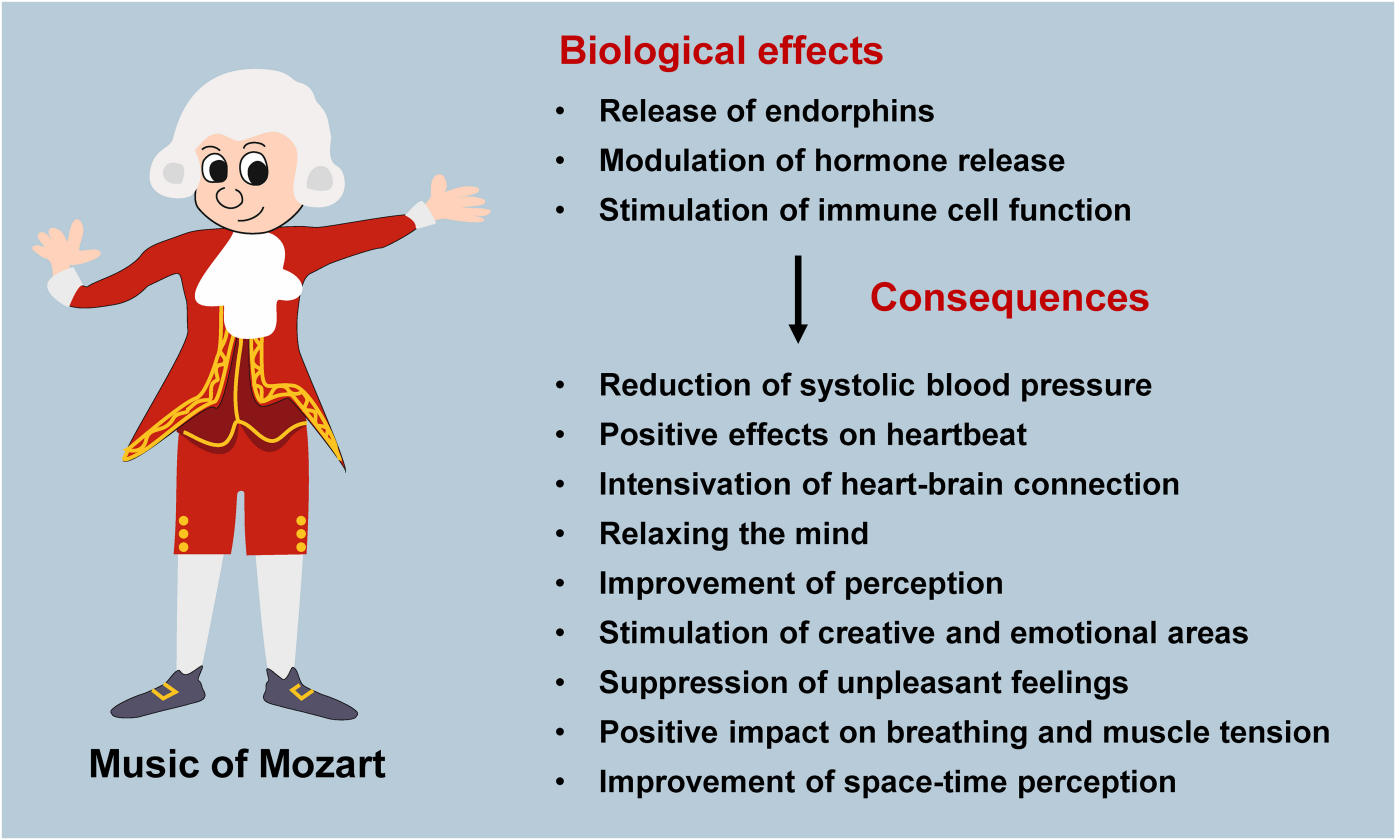
Proposed biological effects and consequences resulting from listening to the music of Mozart. Clinical studies have shown that melodies of Mozart have an impact on the synthesis and release of endorphins and hormones, while also stimulating immune cell function. The resulting consequences are numerous, particularly affecting the cardiovascular system and specific brain regions.

Why Mozart? His tunes relax the mind, improve perception, activate communication between the mind and heart, and stimulate creative and emotional areas. His music also covers unpleasant feelings, equalizes brain waves, and stabilizes blood pressure, pulse as well as heart rate. It influences breathing and reduces muscle tension, increases endorphins, modulates hormonal release, stimulates immune function, and improves space-time perception (Figure 1) (10). Furthermore, it has a simple and unique harmonious structure that can be shared in all countries of the world (11).

Are there alternatives to Mozart? Clearly, yes; evidence suggests that similar effects can be observed after listening to music from various genres, origins, and meanings (12). From the sacred tradition of Gregorian chants to Baroque compositions, Pachelbel's Canon is regarded as an exemplary model for synchronizing brain waves with musical frequencies (4).

The classical symphony with Beethoven's Moonlight Sonata at the forefront, and romantic music, like Schubert's compositions, remain an enchanting standards of beauty. Impressionist music, such as Stravinsky and the Russians, has great evocative effects, especially due to its connection with figurative art. Opera showcases a unique collection of arias, with Rossini being a standout, and songs with unforgettable melodies from our own country.

Changing continents and eras, South American music is often paired with dancing to enhance the atmosphere at social gatherings. Reggae, with its backbeat rhythm, could have specific applications in neuropsychology. Eventually, we arrive at the most influential rock genre, particularly the English progressive genre, which has attempted to replicate the impact of classical music. This involves listening in silence within environments of remarkable natural or historical beauty.

Finally, even pop, although apparently not very suitable (11), has a current like ambient music, which has been involved since the end of the 80s in creating relaxing atmospheres to combat stress (airport music). Music therapy is a form of therapy that uses music to help people improve their physical, emotional and social well-being. It can be used to help people (13).

Naturally, we are particularly interested in the use of music in the context of heart disease. Therefore, the presence of “music medicine” in our lives has become an appealing clinical tool in healthcare settings. Consequently, it is evident that musicians and music therapists share a similar profession that aids in improving health outcomes, especially for cardiovascular diseases.

While discussing therapeutic benefits through listening experiences is essential for understanding how music aids heart patients in relaxation and stress relief; it is equally important to recognize how songwriting parallels academic research processes in fostering creativity and emotional expression relevant to health outcomes.

## 3 Exploring the impact of music therapy across the lifespan

The relationship between music therapy and age is significant, as individuals experience different developmental stages throughout their lives. For children, music therapy can support language development, emotional expression, and social skills. It provides a playful environment where young clients can explore their feelings and build prosocial skills with others through shared musical experiences (14, 15). Music therapy is also valuable for children with kidney and gastrointestinal diseases, particularly during hospital stays (16). Similarly, beneficial effects from music were reported in children during the postoperative period of heart surgery (16). It further lowers the heart rates of severely sick children undergoing haemotopoietic stem cell transplants and reduces pain, fear, and anxiety in children during cardiac catheterization (18, 19). In adolescence, music often becomes a crucial outlet for self-expression, identity formation, and definitions of shared interests with others (20, 21). Music therapy can assist teenagers in navigating the complexities of this life stage by offering them important tools to cope with stressors such as peer pressure, academic demands, and mental health challenges such as anxiety and depression (22). For adults, particularly those facing chronic illness or mental health issues, music therapy can serve as a means of relaxation and emotional release. It fosters resilience by helping individuals process their experiences and emotions through creative expression, providing a robust and positive link with mental health (23).

As people age into their senior years, music therapy continues to play an essential role in enhancing quality of life. It can stimulate memory recall and cognitive functions in individuals with dementia or Alzheimer's disease while promoting social interaction among older adults in group settings (24, 25).

Overall, music therapy is a versatile intervention that adapts to the unique needs associated with each life stage. Its ability to bridge generational gaps makes it an invaluable resource for fostering connection and healing across all ages. However, it should be noted that the effects of different music styles can vary significantly across age groups. Younger individuals may respond more strongly to certain genres, such as Meditative Binaural Music, experiencing greater emotional and physiological relaxation compared to older adults (26). Conversely, older age groups often find traditional forms of music, like calm classical pieces, to be more comforting and soothing. These differences highlight the importance of considering age when evaluating the impact of music on relaxation and emotional well-being (26). In line, a study investigating age-related differences in cardiovascular-autonomic responses to “relaxing” and “aggressive” music among young (average age 22.8) and older (average age 61.7) healthy listeners showed that during silence, older participants exhibited lower autonomic modulation but higher blood pressure compared to younger individuals (27). When exposed to music onset, “relaxing” music decreased heart rate variability in older listeners while increasing systolic blood pressure in younger ones. In contrast, “aggressive” music led to a decrease in heart rate variability and an increase in sympathetic activity for older participants, whereas younger listeners experienced increased systolic blood pressure but decreased parasympathetic activity (27). Overall, the findings suggest that responses to music are influenced by age, with distinct patterns of sympathetic and parasympathetic balance observed between the two groups during music exposure.

It should be critical noted that some of the observed differences in cardiovascular-autonomic responses to music between younger and older individuals may be attributed to the fact that older adults are generally more likely to have underlying heart conditions, which can influence their physiological reactions. Understanding these variations can help tailor music interventions to better suit the needs of different age demographics.

## 4 Musicians and researchers: two similar professions

The career pathways of musicians and researchers/clinicians share several similarities, including the necessity for extensive education and training, dedication to continuous improvement, and the importance of networking within their respective fields. Both musicians and researchers often start with foundational education. Musicians may attend music schools or conservatories, while researchers typically pursue advanced degrees in their disciplines. However, their paths diverge significantly in focus and outcomes: musicians often seek performance opportunities, composition work, or teaching roles, emphasizing creativity and artistic expression. In contrast, researchers and clinicians are driven by the pursuit of knowledge and practical applications in health or science, leading to careers in academia, clinical practice, or industry research. Ultimately, while both careers require passion and commitment, they cater to different talents and societal contributions. However, the intersection of these two professions is beautifully exemplified in music therapy, where musicians collaborate with researchers and clinicians to utilize the therapeutic power of music in promoting mental and physical well-being, thereby enhancing patient care through a unique blend of artistry and scientific understanding.

High-quality music is typically structured in a manner that facilitates memorization. Moreover, humans remember music far better than other stimuli because of repletion, emotional content, and the fact that music is a highly structured stimulus (27). As a result, most successful songs contain simple chords and lyrics. Paul McCartney mentioned that his song “Dance Tonight”, which opened the album “Memory Almost Full”, almost wrote itself due to its simplicity (29). There are various approaches to songwriting, but there are some general necessities and workflows to follow (30, 31). Firstly, the artist must brainstorm and define the subject of the song. In the second step, the lyrics and the melody need to be finalized. After that, the presentation style and instructions for the musicians working with the songwriter must be determined. At this point, the song is considered complete and the musician has achieved their goal. However, in most cases, the artist and band members want to make their song popular and earn money. This requires finding a music company willing to promote the song. Even if the song is enjoyable to listen to, there may be obstacles. The style and chorus must align with the current “Zeitgeist” or spirit of times (32). Songs about war and anti-war, such as “War” by Edwin Starr, “War Pigs” by Black Sabbath, “Gimme Shelter” by The Rolling Stones, “Give Peace a Chance” by the Plastic Ono Band, or “Zombie” by The Cranberries, not only have excellent musical quality but were also released during times of conflicts and crises.

**Figure 2:**
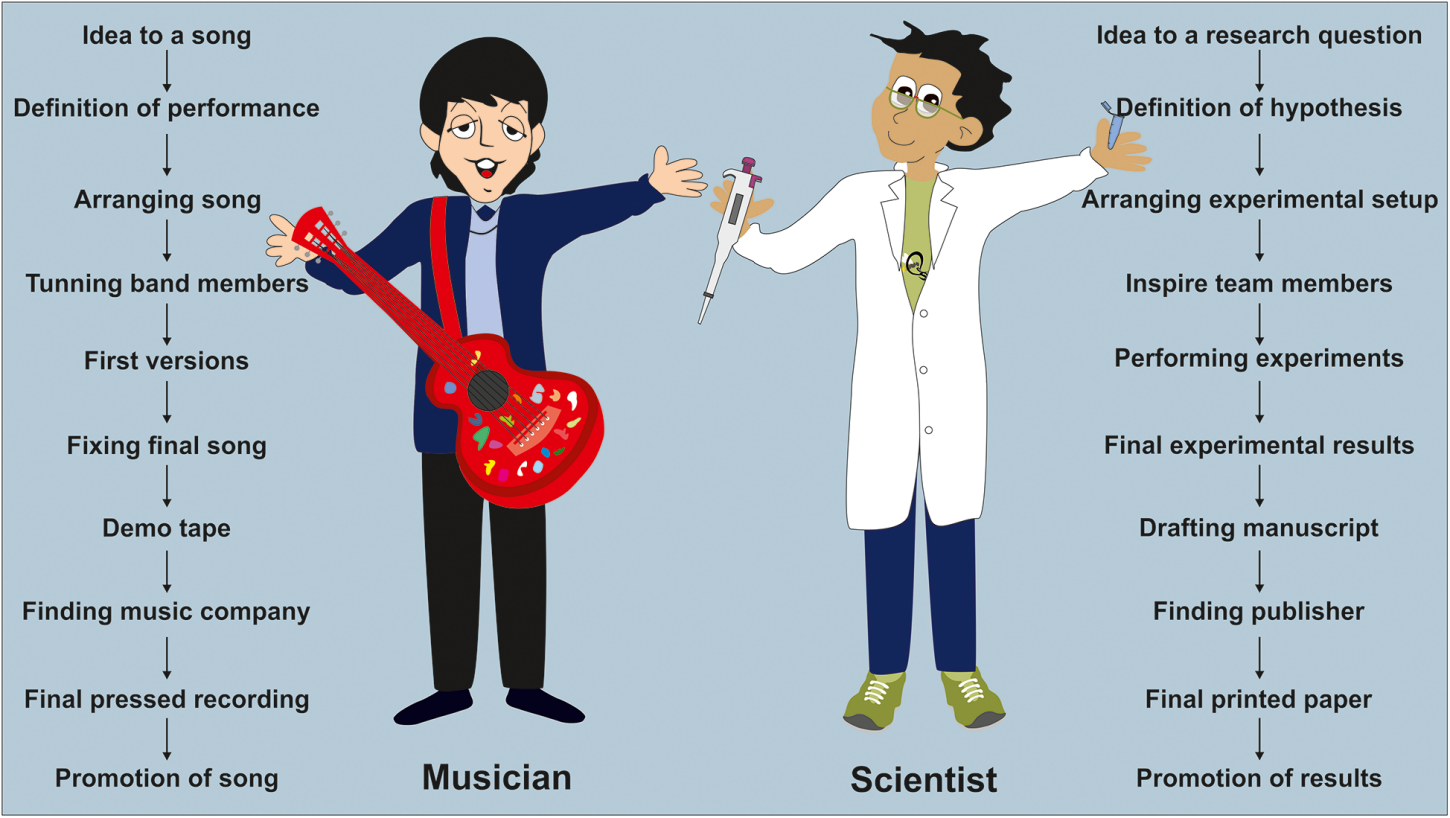
Steps for setting up a song or research paper. There are several necessary steps involved in the process of creating a song or research project. For a musician, these steps include arranging the song, tuning band members, finalizing the composition, convincing a music company to release the song, and promoting it through concerts. Similarly, for a scientist, the steps involve planning experiments, defining a working hypothesis, inspiring team members, conducting experiments, and summarizing findings in a paper. This paper is then printed by a suitable publisher and promoted at national and scientific meetings.

In addition, the release of a song can be restricted or even prevented if the lyrics contradict common norms. A prominent example is the legendary album “Legalize it” by Peter Tosh, which was released in 1976. This album was banned in many countries due to its title track of the same name. In Germany, both the album and the later single release from 1978 “Get up, Stand up”, which had the controversial song “Legalize it” on the B-side, were banned by the national inspection authority for youth-harming media in 1980. The ban was primarily due to the trivialization of drug use (33). The 13-page decision to ban the album included a translation of the song's lyrics into German and scientific evidence of the negative health effects of drug consumption (34). Given Germany's current drug policy, which aims to legalize marijuana for personal use by the middle of 2024, the previous ban seems completely exaggerated from today's perspective. Conversely, the message of the song has gained even more relevance over time. Similar to musicians, scientists also have comparable workflows in their daily routines (Figure 2).

First, he needs to think about an interesting research project that is worth designing and investigating (35). In order to succeed the project should align with his skills and interests. After formulating the research questions, he must consider the methods, tools and models that will be used to address these questions (35). These elements can be compared to the cords, strings, and wording that an artist must find for their song. Once the correct experimental setup is determined, the researcher must then organize the sequence and repetitions of the experiments and instruct their team members (Postdocs, PhDs/MDs candidates, bachelors, masters, technicians) accordingly. If the researcher is fortunate, they and their team can draw scientific conclusions provide answers that may be of interest to the public or those working in their research area. However, the most critical part comes next—finding a publisher willing to publish the findings (36), which are summarized in a manuscript (the “researcher's song”). Similar to an artist who relies on the decision makers at a music company, the researcher must persuade journal editors and reviewers that their findings are worth publishing. Unfortunately, at this stage, the findings usually don't go directly to press. In most cases, if the paper is not rejected, the researcher is asked to add more chords or words, or even write completely new verses, which means conducting additional experiments to support their findings.

However, things can get worse when reviewers propose experiments and request additions that have nothing to do with the original project. It's like taking a part of “Zombie” by The Cranberries and adding it to Paul McCartney's “Dancing tonight”. As a result, many researchers try to avoid incorporating of “wrong tones” forced by these “dangerous assessors”. One simple way is to publish findings in pay-to-publish journals, where researchers pay fees to get their work printed. Some researchers believe this is akin to buying their way to the top of the charts, even if their work lacks credibility and scientific soundness. However, these “findings” are then accessible online to a wide audience. Another way to simplify the publication process is if the researcher has good relations with editorial board members, or even better, with a journal editor. In some journals, board members can “communicate” one or more papers per year in their own area of expertise. While this is often denied, having good “scientific” relationships with influential scientists can certainly be advantageous when trying to publish research results (37).

Nowadays, scientists have found a third way to simplify publication. The establishment of “Preprint servers” allows us to publish papers without any review process (38). Actually, the “Accelerating Science and Publication in Biology (ASAPbio)” represents a scientist-driven nonprofit working initiative aiming to drive open and innovative communication in life sciences, and it lists 65 of these preprint servers (as of November 29, 2024) (39). Among them are AAS Open Research, Authorea, bioRxiv, ChemRxiv, F1000 Research, Preprints.org, Research Square, and Zenodo, which allow for fast and wide spreading of research results without a review process. Interestingly, some publishers of regular journals also offer authors the option to deposit their findings onto preprint servers. Moreover, some journals allow the citation of preprints. However, preprint articles are not peer-reviewed, so it is difficult to determine if the work is good or not, unlike a music song. Similar to an artist, a researcher can increase the probability of publishing their findings if they are working on a “hot research topic” or a new technology that represents a kind of “Scientific Zeitgeist” (40).

The worldwide COVID-19 pandemic for example had a significant impact on the number and topics of publications (41). There was an exponential rise in COVID-19 publications, while the number of non-COVID research articles decreased significantly (41). This can be compared to the phenomenon of “Beatlemania”, which was the euphoria surrounding The Beatles. It began on October 13, 1963 when the band first performed on the TV show Val Parnell's Sunday Night at the London Palladium, reaching an estimated audience of up to 15 million people (42). Subsequently, the frequency of Beatles songs played on radio stations increased significantly.

Finally, the quality of a musician and a researcher is measured in similar ways. These measures include the number of record sales, rankings on music charts, cumulative digital downloads and streams of songs from music portals, and visitor numbers at concerts. Similarly, the quality of a researcher is estimated by the number of publications, cumulative impact factor, citations, talks given at national or international meetings, and memberships to scientific organizations and journal management teams (43, 44). This is where the cat bites its own tail. Is it possible that a song performed by a specific musician is ranked higher if they have previously performed excellent songs? Has anyone heard of McCartney writing a bad song? Is it easier for a researcher to publish a paper when they already have a high reputation?

## 5 Music in science and science in music

Music listening inspires scientists in their daily work, and music therapy, which utilizes musical elements under the guidance of trained and qualified therapists, is being studied and utilized in clinical settings (45–47). Our human connection to music is deeply rooted in our biology, and this connection extends to laboratory and farm animals, who also exhibit physiological and behavioral changes when exposed to music (48–50). For example, mice offspring whose mothers listened to music for 2 h a day during pregnancy showed improvements in ambulation score, grip strength, and limb suspension (51). Additionally, both listening to and playing music can lead to epigenetic alterations by influencing the expression of genes and miRNAs (52). Music therapy sessions have been found to effectively in reduce anxiety and distress in non-small cell lung cancer survivors undergoing immunotherapy (53). Furthermore, engagement with music has shown to mitigate the harmful effects of COVID-19 infections, particularly in relation to inflammation and blood clotting pathways that are associated with pathophysiological and neuropathological issues (54). However, it is important to note that music therapy does not work for all diseases. A recent multicenter randomized clinical trial found that music intervention did not effectively decrease anxiety in adult critically ill patients (55). Similarly, it is unlikely that someone would find relief from a headache by listening to loud hard rock music. Therefore, it appears that there are specific areas in which music can be therapeutically effective.

Undoubtedly, in patients with heart disease, music serves as a therapeutic tool that promotes relaxation by reducing stress levels. As discussed, listening to calming melodies can lower cortisol levels (56), thereby alleviating tension within the body. This reduction in stress not only helps patients feel more at ease but also positively influences cardiovascular functions such as lowering heart rate and blood pressure. Moreover, engaging with soothing music can create a meditative state that fosters emotional healing (57, 58). As patients immerse themselves in musical experiences—whether through passive listening or active participation—they may find relief from anxiety related to their condition. Ultimately, incorporating music into treatment plans offers a holistic approach that addresses both the psychological well-being of heart disease patients while supporting their physical health outcomes.

Musicians may find inspiration in these scientific findings as they highlight the profound impact of music on health, particularly for patients with heart disease. Understanding that their art can serve as a therapeutic tool can motivate musicians to create calming melodies specifically designed to promote relaxation and emotional healing. In particular, the supportive role of music-making was demonstrated during the COVID-19 pandemic, in which it was found to prevent or limit the onset of depression or more serve forms of mental illness (59). This knowledge encourages them to explore the therapeutic potential of their compositions, recognizing that their music can help alleviate stress and improve cardiovascular function. Additionally, the idea that engaging with music can foster a meditative state may inspire musicians to incorporate elements of mindfulness into their performances or songwriting processes, ultimately enhancing the emotional connection between their music and listeners seeking relief from anxiety or discomfort related to health challenges.

Therefore, musicians can be seen, to some extent, as doctors without doctoral degrees. In fact, there are musicians who have earned PhD or MD degrees. Brian May, the guitarist of Queen, obtained his doctorate in astrophysics in 2007 for his work on “Radical Velocities in the Zodiacal Dust Cloud”. He has also been honored with a Doctorate of Science degree from the University of Hull in England (60). Similarly, Dr. Alban, a Nigerian-Swedish musician and producer, holds a doctorate in dentistry. Mira Aroyo, the keyboardist and singer of the British band Ladytron, completed her studies at Oxford and earned a doctorate in molecular genetics (61, 62).

Similarly, musicians can benefit from the innovations made in research. This was recently highlighted in a BBC radio interview with Paul McCartney, where he revealed that he has utilized artificial intelligence in the creation of the final Beatles record that was released at the end of 2023 (63). While overdub remixes and cleaned-up versions of the song “Now and Then”, originally recorded by John Lennon in 1977, have already been circulating on various internet platforms (such as YouTube) and unofficial bootleg releases, the use of artificial intelligence has enabled the extraction and purification of Lennon's voice. This breakthrough has served as the foundation for the final unique Beatles track.

Moreover, the worldwide music production of music and the evaluation of music quality using deep learning techniques have become major areas of interest in recent years (64–66). There are both advantages and disadvantages to the creation and validation of music by artificial intelligence. On one hand, melodies, chords and lyrics can be easily established and combined, and artificial intelligence can serve as a source of musical inspiration. On the other hand, the authenticity and human touch of a song may be lost. In addition, there may be legal issues that need to be considered.

In this context, the music industry can potentially learn from the use of artificial intelligence by researchers who have employed cheating research paper mills and “scientific” on-demand publishers. These profit-oriented organizations write and sell fraudulent research papers that closely resemble genuine research. These factories of fake paper have already produced thousands or even tens of thousands of papers that contaminate the literature and undermine the credibility of the entire scientific community (67). Unfortunately, the number of papers originating from paper mills is increasing, resulting in negative consequences for the scientific community (68). Consequently, many journals have implemented strategies to identify such papers (69). It is interesting that now artificial intelligence is being employed to detect papers in which text- and image-generating software has produced fraudulent data, as well as to address the growing problem of paper mills. Furthermore, it is necessary to distinguish between papers written with the assistance of artificial intelligence and those that have been completely fabricated (70). It appears that scientists are using the “fight fire with fire” approach.

Likewise in music production, AI is now also an important tool for improving music therapies. One study established the main predictive factors for the relaxation effects of music listening using machine learning methods (71). The identified decision tree with an overall accuracy of 0.79 makes it possible to find predictive factors that impact therapeutic music listening outcomes (71). Another study explored the integration of music therapy and AI by developing a machine learning model to assist music therapists in selecting appropriate music for patients (72). The AI system uses a multi-class neural network to classify emotions and predict the therapeutic benefits of specific songs based on individual musical preferences and emotional characteristics. This study demonstrates how AI can enhance the practice of music therapy by providing personalized recommendations, thereby improving treatment outcomes for patients (72). Moreover, there are efforts to analyze music therapy using AI methodologies to enhance its effectiveness (73).

Nowadays, society has recognized the deep connection between music and science. Consequently, SAGE Publications Ltd. launched the journal “Music & Science” in 2018. This peer-reviewed journal provides a platform for researchers to communicate important new insights into the Philosophy of Science, Music, and Psychology, highlighting the significant overlap between these disciplines (74). Similarly, Oxford Academic has launched the “Journal of Music Therapy”, which promotes scholarly activity in music therapy and fosters the development and understanding of music therapy and music-based interventions (75). Additionally, this publisher prints “Music Therapy Perspectives”, which focuses on the clinical benefits of music therapy (76). Likewise, the journal “Music and Medicine” published by the International Association for Music & Medicine, serves as an integrative forum for clinical practice and research related to music interventions and the application of clinical music strategies in medicine (77).

## 6 Music-based therapies

**Figure 3:**
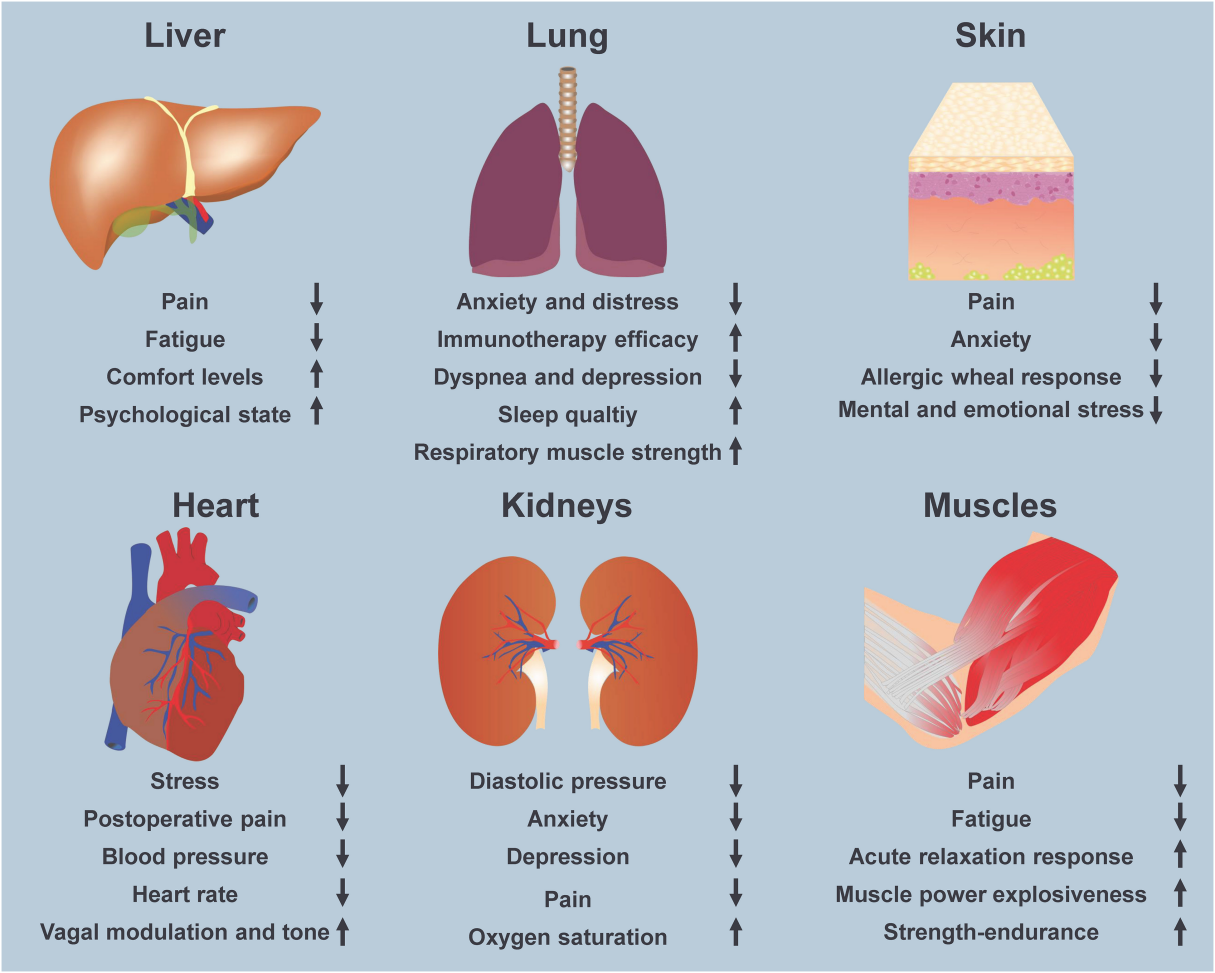
Music in the treatment of human disease. Music and music-directed therapies have been successful in treating a wide range of physical and mental ailments. Beneficial effects on various organs, including the liver, lungs, skin, heart, kidneys, and muscles have been reported.

Screening the PubMed database for the search term “Music and therapy” yielded a total of 15,513 publications (search conducted on November 29, 2024). Among the initial results featuring these terms are “Cure by music” (78), “Music in the post-war therapy” (79), “Music and the mentally ill” (80), and “Music and occupational therapy” (81), all suggesting the therapeutic potential of music. A search of the PubMed database revealed a substantial body of research on music's therapeutic potential on heart health, indicating significant interest in its impact on cardiovascular care (see above). This suggests that music plays a valuable role in both general therapeutic practices and specific heart treatments, warranting further exploration. However, it is widely recognized that music has beneficial effects not only on cardiovascular issues but also has the capacity to mitigate symptoms in many other organs (Figure 3).

In the clinical setting, music has been found to have highly beneficial effects on human health. Specifically, it reduces the stress hormone cortisol (56), boosting endorphin production for improved mood (10), enhancing focus and motivation (82), alleviating physical pain (83), especially chronic pain, lowering blood pressure, and improving well-being, which support overall cardiovascular function (Table 1). It also promotes relaxation, reduces stress, improves sleep quality, and can aid in recovery from injury or illness by enhancing coordination, strength, and flexibility. Furthermore, music therapy is beneficial for various disorders such as depression, anxiety, and dementia (13).

**Table 1:** List of the beneficial effects of music on health.

Effects/Potential operational area of music	Cause	Reference
Reduces stress	Listening to music can reduce levels of cortisol, which is the stress hormone.	(56)
Improves mood	Music can activate the production of endorphins and natural chemicals that improve mood.	(10)
Increases motivation and concentration	Listening to music can help individuals become more focused and stay motivated.	(82)
Helps reduce pain	Music can help reduce physical pain, particularly chronic pain.	(83)
Usage in hospitals and clinics	Music can be used to help patients relax and reduce stress. Additionally, it can aid in improving patients’ sleep quality and enhancing their tolerance towards medical treatments.	(57, 58)
Usage in rehabilitation	Music can be utilized as a means to aid patients in their recovery from injury or illness. It has the potential to enhance coordination, strength, and flexibility.	(84)
Usage in therapy	Music can be used as therapy for a variety of disorders, including depression, anxiety, and dementia.	(13)

It counteracts anxiety in dementia patients and decreases agitation in Alzheimer's disease (85). Similarly, music interventions promote better diabetic conditions by reducing blood sugar, lowering stress and heart rate, thereby enhancing the psychological and physical health of patients with diabetes (86). In liver disease, it reduces intensity of pain in patients with end-stage liver disease (87) and improves the psychological state in patients with acute liver failure (88). Similarly, music decreases pain and anxiety in many skin diseases including psoriasis, neurodermatitis, atopic dermatitis, and contact eczema, most likely by affecting cytokine levels or allergic wheal response (83). In regard to the kidneys, a two to four times a week therapy with music has shown highly beneficial effects in children suffering from nephrological diseases (89). In addition, muscle tension levels are improved in patients with emergent medical conditions (90). Highly beneficial effects have also been found in patients with hypertension and various cardiovascular diseases (91–93). Finally, future controlled trials are being planned to prove the efficacy of music in the therapy of respiratory conditions (94).

Studies on the effects of music on the heart in both healthy individuals and patients with heart diseases have often produced inconsistent results. This is likely due to the use of varied methods, measurements, types of music across different studies, and stimuli (e.g., loudness, genre, instrumentation, and tempo) across studies (95). However, there has been a significant increase in interest from scientists and clinicians in understanding the impact of music on heart parameters in recent decades. A search on PubMed using the keywords “heart and music” yielded 1,920 hits (search conducted on November 29, 2024). A recent single-blinded controlled trial found that listening to nature-based music while in a prone position reduced anxiety and improved oxygenation and heart rate in conscious and hypoxemic COVID-19 patients (96). In line, a meta-analysis of 20 randomized controlled studies (RCTs) involving 2,306 patients with hypertension, of which 1,154 received music therapy, showed that music is effective in reducing blood pressure, heart rate, anxiety, and depression. Additionally, it was found to improve sleep quality (91).

Music can be a powerful tool for helping patients with heart disease relax and relieve stress, which is crucial for their overall cardiovascular health (97). By listening to calming music, a reduction in cortisol levels leads to a more tranquil state of mind (98). This relaxation response not only helps lower blood pressure but also reduces heart rate, creating a more favorable environment for healing. Additionally, engaging with music can shift focus away from anxiety and discomfort, allowing individuals to experience moments of joy and emotional release. As patients incorporate music into their daily routines—whether through passive listening or active participation—they may find it easier to manage the psychological challenges that accompany heart disease, ultimately fostering a sense of well-being that supports their recovery and long-term health (99, 100).

While the therapeutic benefits of music on cardiovascular health and other clinical measures are well-documented, several factors may lead to inconsistencies in outcomes. One significant factor is the variability in music selection (26). The type of music chosen for therapy can greatly influence its effects on cardiovascular parameters. Different genres, tempos, and rhythms may elicit varying emotional responses and physiological reactions; for instance, calming music might lower heart rate and blood pressure, while more energetic music could have the opposite effect. Individual preferences also play a crucial role. What is soothing for one person may be stressful for another (101). This variability complicates the establishment of standardized protocols for using music as a therapeutic intervention.

Additionally, patient heterogeneity introduces further complexity. Patients differ widely in their medical histories, psychological states, and personal experiences with music. Factors such as pre-existing cardiovascular conditions, mental health status, and previous exposure to music therapy can affect how individuals respond to musical interventions. This diversity makes it challenging to generalize findings across populations or predict outcomes based on specific musical selections.

The age of patients is another important consideration that can contribute to inconsistent effects of music on cardiovascular health. Age-related differences mean that older adults may have different physiological responses to music compared to younger individuals due to changes in heart function, vascular elasticity, and overall health status. Moreover, older adults might have distinct musical preferences shaped by cultural contexts that could influence their engagement with the therapy.

Other relevant factors also come into play. For example, the environment in which the music is played, whether in clinical settings or at home, can potentially impact its effectiveness. The duration and frequency of exposure to music therapy sessions may also alter outcomes significantly. Furthermore, individual psychological traits such as anxiety levels can interact with the effects of music on cardiovascular health.

In conclusion, while there is substantial potential for music therapy to positively impact cardiovascular health, these inconsistencies underscore the need for personalized approaches that consider individual differences in musical preference and context. Thorough research into how various factors interact with therapeutic outcomes will be essential in optimizing the use of music as a treatment modality.

In pediatric patients with kidney failure requiring hemodialysis, live music significantly reduced heart rate, systolic pressure and diastolic pressure (102). Similarly, a randomized controlled trial demonstrated that music is effective in lowering dental anxiety and fear, heart rate, and blood pressure, while increasing oxygen saturation levels (103). Furthermore, a recent review showed that music intervention is a beneficial element in managing diabetic conditions by improving patients' engagement in exercise, autonomous balance, and lower limb blood circulation (86). Similarly, music therapy resulted in reduced heart rates, breathing rates, and discomfort levels in patients in a pediatric intensive care unit (104). Beneficial effects of music were also proven in patients who underwent interventional cardiac catheterization, in which music listening preceding the intervention reduced anxiety and stress response (105).

Consequently, experts propose utilizing music during bedside procedures in intensive care units, as music has been found to affect important physiological parameters such as blood pressure, respiratory rate and heart, while also lowering subjective pain and anxiety in patients (106). However, it is possible that music has relaxing and calming effects on employees working in these specialized medical and nursing care units, indirectly alleviating patient fear. Furthermore, music may serve as a distraction, helping patients to focus less on their illness and potentially provoking a positive systemic effect. Overall, these potential effects of music make it therapeutically valuable.

Nevertheless, there is also data showing that music does not necessarily influence the cardiovascular system. A meta-analysis conducted in accordance with PRISMA guidelines and including data from 25 articles found that music sessions reduced anxiety levels but did not have an impact on systolic/diastolic pressure, respiratory rate, or heart rate (107).

Therefore, there is still a need for more systematic studies to investigate the beneficial effects of music on the heart. Unfortunately, the methodological recommendations published by Koelsch and Jäncke in 2015 were widely ignored (95). It is important to clearly define musical and acoustical features, as well as the musical preferences of healthy and diseased patients included in the studies. Additionally, psychologically and economically relevant outcome measures should be clearly defined to allow for better comparability between individual studies.

## 7 Conclusion

**Figure 4:**
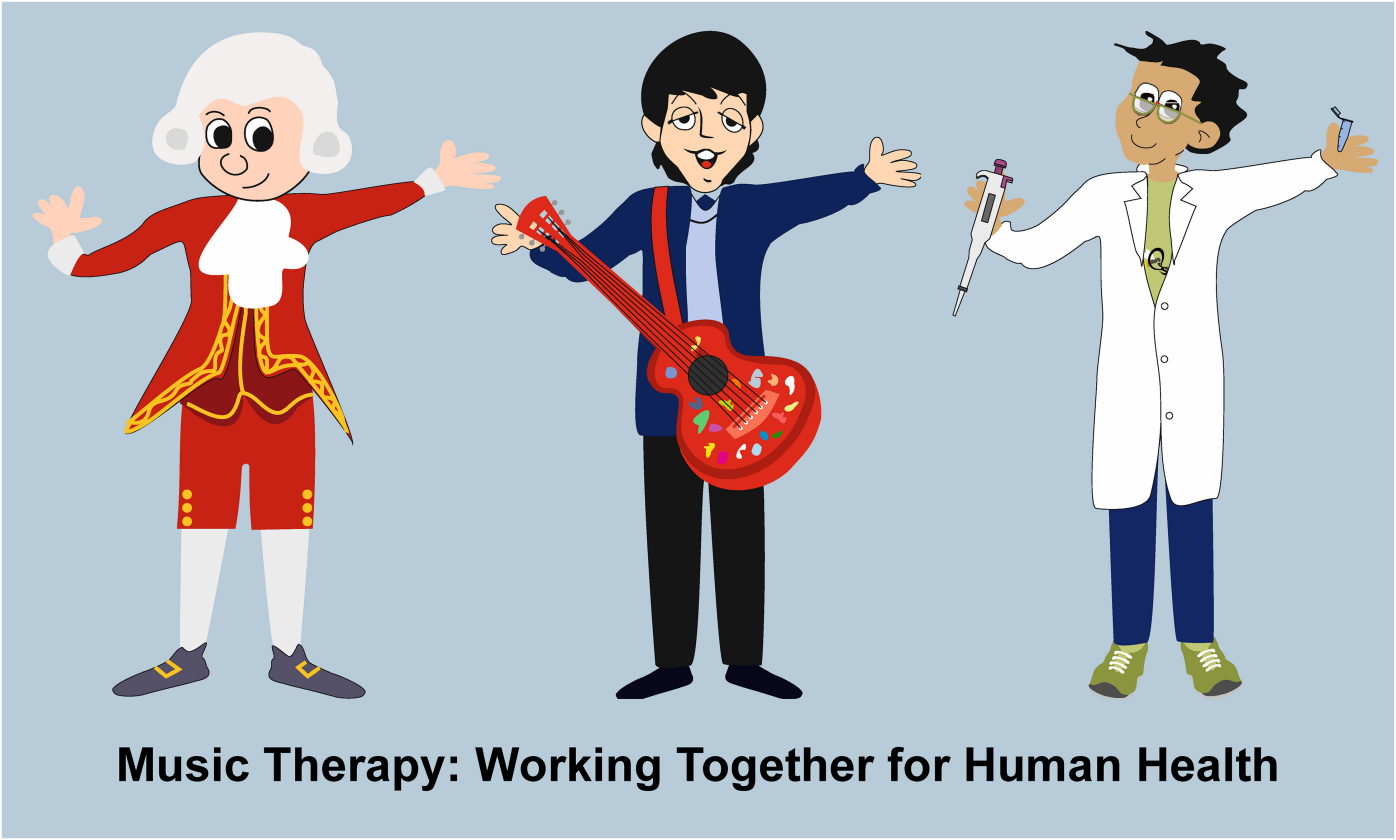
Graphical abstract. Musicians and researchers play vital roles in promoting human health. The interconnectedness of these two professions emphasizes the importance of interdisciplinary efforts in enhancing well-being and advancing health initiatives through creating suitable music (musicians) and conducting research or music therapy (researchers/clinicians).

In conclusion, musicians and researchers share many similarities in their professions. Their work requires creativity and commitment to continuous improvements, such as the introduction of AI in their daily routine works. Whether they are conducting experiments or composing a symphony or song, musicians and researchers are united in their pursuit of knowledge and their desire to share their discoveries and musical talents with the world. Music has long been recognized as a powerful tool in therapy that can evoke emotions, stimulate memories, improve emotional well-being, increase self-awareness, and enhance mental well-being. Studies have shown that music therapy holds promise in the treatment of heart conditions by improving cardiovascular function and reducing stress levels. Musicians, researchers, and clinicians are essential professionals who stand in line and contribute to human health through writing music or performing music therapy (Figure 4). However, music therapy also has some potential disadvantages and side effects, such as emotional distress when certain songs trigger negative feelings. Cultural sensitivity is important, as musical preferences vary widely, which may limit effectiveness for some individuals. Therefore, there is an urgent need to conduct high-quality clinical trials to establish evidence-based practices in music therapy, rather than relying on anecdotal cases like that of Little Krissy. Further research is needed to fully understand the mechanisms underlying its effectiveness and to determine the optimal dosage and duration of music therapy sessions for the treatment of different diseases.

## References

[1] Thoma MV La Marca R Brönnimann R Finkel L Ehlert U Nater UM. The effect of music on the human stress response. PLoS One. (2013) 8(8):e70156. 10.1371/journal.pone.0070156 23940541 PMC3734071

[2] Pauwels EK Volterrani D Mariani G Kostkiewics M. Mozart, music and medicine. Med Princ Pract. (2014) 23(5):403–12. 10.1159/000364873 25060169 PMC5586918

[3] Sleight P. Music and the heart. Eur Heart J. (2015) 36(28):1786. 26413596

[4] Campbell D. L’effetto Mozart. Curarsi con la musica. Milan: Dalai Editore. (2002).

[5] Darki C Riley J Dadabhoy DP Darki A Garetto J. The effect of classical music on heart rate, blood pressure, and mood. Cureus. (2022) 14(7):e27348. 10.7759/cureus.27348 36046316 PMC9417331

[6] Rauscher FH Shaw GL Ky KN. Music and spatial task performance. Nature. (1993) 365(6447):611. 10.1038/365611a0 8413624

[7] Jenkins JS. The mozart effect. J R Soc Med. (2001) 94(4):170–2. 10.1177/014107680109400404 11317617 PMC1281386

[8] Campbell G. The Mozart Effect: Tapping the Power of Music to Heal the Body, Strengthen the Mind, and Unlock the Creative Spirit. 1st ed. New York: Avon Books (1997).

[9] Giannouli V Yordanova J Kolev V. Can brief listening to Mozart’s music improve visual working memory? An update on the role of cognitive and emotional factors. J Intell. (2024) 12(6):54. 10.3390/jintelligence12060054 38921689 PMC11204774

[10] Dunbar RI Kaskatis K MacDonald I Barra V. Performance of music elevates pain threshold and positive affect: implications for the evolutionary function of music. Evol Psychol. (2012) 10(4):688–702. 10.1177/147470491201000403 23089077 PMC10426978

[11] Gruhlke LC Patrício MC Moreira DM. Mozart, but not the beatles, reduces systolic blood pressure in patients with myocardial infarction. Acta Cardiol. (2015) 70(6):703–6. 10.1080/AC.70.6.3120183 26717219

[12] Dileo C. The present and future of medical music therapy for adults in the U.S.A. J Urban Cult Res. (2021) 23:79–93.

[13] AIM. Musicoterapia. (2024). Available online at: https://www.aim-musicoterapia.it/musicoterapia (accessed December 21, 2024).

[14] Blasco-Magraner JS Bernabe-Valero G Marín-Liébana P Moret-Tatay C. Effects of the educational use of music on 3- to 12-year-old children’s emotional development: a systematic review. Int J Environ Res Public Health. (2021) 18(7):3668. 10.3390/ijerph18073668 33915896 PMC8037606

[15] Wu X Lu X. Musical training in the development of empathy and prosocial behaviors. Front Psychol. (2021) 12:661769. 10.3389/fpsyg.2021.661769 34045996 PMC8144324

[16] Kobus S Buehne AM Kathemann S Buescher AK Lainka E. Effects of music therapy on vital signs in children with chronic disease. Int J Environ Res Public Health. (2022) 19(11):6544. 10.3390/ijerph19116544 35682129 PMC9180355

[17] Hatem TP Lira PI Mattos SS. The therapeutic effects of music in children following cardiac surgery. J Pediatr. (2006) 82(3):186–92. 10.2223/JPED.1473 16680285

[18] Uggla L Bonde LO Svahn BM Remberger M Wrangsjö B Gustafsson B. Music therapy can lower the heart rates of severely sick children. Acta Paediatr. (2016) 105(10):1225–30. 10.1111/apa.13452 27129139

[19] Karakul A Akgül EA Yalınız R Meşe T. Effectiveness of music during cardiac catheterization on children’s pain, fear, anxiety and vital signs: a randomized, blind controlled trial. J Pediatr Nurs. (2022) 65:e56–62. 10.1016/j.pedn.2022.02.009 35279331

[20] Chen L. Influence of music on the hearing and mental health of adolescents and countermeasures. Front Neurosci. (2023) 17:1236638. 10.3389/fnins.2023.1236638 37600009 PMC10434992

[21] Uhlig S Jansen E Scherder E. “Being a bully isn't very cool…”: Rap & Sing music therapy for enhanced emotional self-regulation in an adolescent school setting - a randomized controlled trial. Psychol Music. (2018) 46(4):568–87. 10.1177/0305735617719154 30369705 PMC6187249

[22] Rodwin AH Shimizu R Travis R Jr James KJ Banya M Munson MR. A systematic review of music-based interventions to improve treatment engagement and mental health outcomes for adolescents and young adults. Child Adolesc Social Work J. (2023) 40:537–66. 10.1007/s10560-022-00893-x PMC9666939 36407676

[23] Jean-Berluche D. Creative expression and mental health. J Creativity. (2024) 34:100083. 10.1016/j.yjoc.2024.100083

[24] Lin TH Liao YC Tam KW Chan L Hsu TH. Effects of music therapy on cognition, quality of life, and neuropsychiatric symptoms of patients with dementia: a systematic review and meta-analysis of randomized controlled trials. Psychiatry Res. (2023) 329:115498. 10.1016/j.psychres.2023.115498 37783097

[25] Bleibel M El Cheikh A Sadier NS Abou-Abbas L. The effect of music therapy on cognitive functions in patients with Alzheimer’s disease: a systematic review of randomized controlled trials. Alzheimers Res Ther. (2023) 15(1):65. 10.1186/s13195-023-01214-9 36973733 PMC10041788

[26] Lee-Harris G Timmers R Humberstone N Blackburn D. Music for relaxation: a comparison across two age groups. J Music Ther. (2018) 55(4):439–62. 10.1093/jmt/thy016 30272211

[27] Hilz MJ Stadler P Gryc T Nath J Habib-Romstoeck L Stemper B Music induces different cardiac autonomic arousal effects in young and older persons. Auton Neurosci. (2014) 183:83–93. 10.1016/j.autneu.2014.02.004 24636674

[28] Levitin DJ Fleming L. Music and memory. In: Kahana MJ Wagner AD, editors. The Oxford Handbook of Human Memory, Two Volume Pack: Foundations and Applications, Oxford Library of Psychology. Oxford: Oxford University Press (2024).

[29] Universal Music Group. “Memory almost full” – track by track. (2024). Available online at: https://www.universal-music.de/paulmccartney/news/memory-almost-full-track-by-track-63910 (accessed December 21, 2024).

[30] Peterik J Austin D Lynn C. Songwriting for Dummies. Hoboken, NJ, USA: Wiley Publishing Inc. (2010).

[31] Rodgers JP. The Complete Singer-Songwriter: A Troubadour’s Guide to Writing, Performing, Recording & Business: A Troubadour’s Guide to Writing, Performing, Recording, and Business. 2nd ed. Edinburgh: Backbeat Records (2016).

[32] Williams M. How a song captures the zeitgeist. (2024). Available online at: https://artists.spotify.com/blog/how-a-song-captures-the-zeitgeist (accessed December 21, 2024).

[33] BPjM. Bundesprüfstelle für jugendgefährdende Schriften. “LEGALIZE IT” Entscheidung Nr 2909 vom 12. Juni 1980, Liste Nr 4246 (Pr. 89/80 und 90/80). Bundesanzeiger, Jahrgang 32. Ausgegeben am Donnerstag, dem 19.6.1980, G1989 AX, page 5 (1980).

[34] Hajok D. Schlaglichter aus 60 jahren bundesprüfstelle. BPJM. (2014) 4:8–18.

[35] Creswell JW Creswell JD. Research Design: Qualitative, Quantitative, and Mixed Methods Approaches. 6th ed. New York: SAGE Publications, Inc. (2017).

[36] Day RA Gastel B. How to Write and Publish a Scientific Paper. 7th ed. Cambridge: Cambridge University Press (2012).

[37] Helgesson G Radun I Radun J Nilsonne G. Editors publishing in their own journals: a systematic review of prevalence and a discussion of normative aspects. Learn Publ. (2022) 35(2):229–40. 10.1002/leap.1449

[38] Smyth AR Rawlinson C Jenkins G. Preprint servers: a ‘rush to publish’ or ‘just in time delivery’ for science? Thorax. (2020) 75(7):532–3. 10.1136/thoraxjnl-2020-214937 32312737 PMC7361021

[39] ASAPbio. List of preprint servers: policies and practices across platform. (2024). Available online at: https://asapbio.org/preprint-servers (accessed December 21, 2024).

[40] Wei T Li M Wu C Yan XY Fan Y Di Z Do scientists trace hot topics? Sci Rep. (2013) 3:2207. 10.1038/srep02207 23856680 PMC3712320

[41] Raynaud M Goutaudier V Louis K Al-Awadhi S Dubourg Q Truchot A Impact of the COVID-19 pandemic on publication dynamics and non-COVID-19 research production. BMC Med Res Methodol. (2023) 21(1):255. 10.1186/s12874-021-01404-9 PMC8607966 34809561

[42] The Beatles Bible. Beatlemania begins: Sunday night at the London palladium. (2024). Available online at: https://www.beatlesbible.com/1963/10/13/beatlemania-begins-sunday-night-at-the-london-palladium/#google_vignette (accessed December 21, 2024).

[43] Shanta A Pradhan AS Sharma SD. Impact factor of a scientific journal: is it a measure of quality of research? J Med Phys. (2013) 38(4):155–7. 10.4103/0971-6203.121191 24672148 PMC3958993

[44] Morrison PJ. The hirsch index and measuring the quality of scientific papers. Ulster Med J. (2008) 77(1):1–2.

[45] Solanki MS Zafar M Rastogi R. Music as a therapy: role in psychiatry. Asian J Psychiatr. (2013) 6(3):193–9. 10.1016/j.ajp.2012.12.001 23642975

[46] Weiskirchen R. The beatles in life sciences: facts and fictions. Biochem Mol Biol Educ. (2022) 50(3):334–44. 10.1002/bmb.21617 35302272

[47] Rabeyron T Robledo Del Canto JP Carasco E Bisson V Bodeau N Vrait FX A randomized controlled trial of 25 sessions comparing music therapy and music listening for children with autism spectrum disorder. Psychiatry Res. (2020) 293:113377. 10.1016/j.psychres.2020.113377 32798927

[48] Alworth LC Buerkle SC. The effects of music on animal physiology, behavior and welfare. Lab Anim. (2013) 42(2):54–61. 10.1038/laban.162 23340788

[49] Taheri F Joushi S Esmaeilpour K Sheibani V Ebrahimi MN Taheri Zadeh Z. Music alleviates cognitive impairments in an animal model of autism. Int J Dev Neurosci. (2023) 83(5):399–416. 10.1002/jdn.10260 37246451

[50] Gao X Gong J Yang B Liu Y Xu H Hao Y Effect of classical music on growth performance, stress level, antioxidant index, immune function and meat quality in broilers at different stocking densities. Front Vet Sci. (2023) 10:1227654. 10.3389/fvets.2023.1227654 37601747 PMC10437118

[51] Bidari S Zendehdel M Hassanpour S Rahmani B. Maternal music exposure during pregnancy influences reflexive motor behaviors in mice offspring. Int J Dev Neurosci. (2023) 83(6):546–51. 10.1002/jdn.10285 37409630

[52] Beccacece L Abondio P Cilli E Restani D Luiselli D. Human genomics and the biocultural origin of music. Int J Mol Sci. (2021) 22(10):5397. 10.3390/ijms22105397 34065521 PMC8160972

[53] Duan X Jia Y Chai J Li W Tang L Li A Music therapy, quality of life and efficacy of immunotherapy for NSCLC. BMJ Support Palliat Care. (2023) spcare-2023-004325. 10.1136/spcare-2023-004325 37673470

[54] Saifman J Colverson A Prem A Chomiak J Doré S. Therapeutic potential of music-based interventions on the stress response and neuroinflammatory biomarkers in COVID-19: a review. Music Sci. (2023) 6:1–18. 10.1177/20592043221135808

[55] Kakar E Ottens T Stads S Wesselius S Gommers DAMPJ Jeekel J Effect of a music intervention on anxiety in adult critically ill patients: a multicenter randomized clinical trial. J Intensive Care. (2023) 11(1):36. 10.1186/s40560-023-00684-1 37592358 PMC10433648

[56] Bradt J Dileo C Potvin N. Music for stress and anxiety reduction in coronary heart disease patients. Cochrane heart group, ed. Cochrane Database Syst Rev. (2013) 2013(12):CD006577. 10.1002/14651858.CD006908.pub2 24374731 PMC8454043

[57] Giordano F Losurdo A Quaranta VN Campobasso N Daleno A Carpagnano E Effect of single session receptive music therapy on anxiety and vital parameters in hospitalized COVID-19 patients: a randomized controlled trial. Sci Rep. (2022) 12(1):3154. 10.1038/s41598-022-07085-8 35210504 PMC8873232

[58] Zanchi B. La musicoterapia e le sue applicazioni nei contesti ospedalieri in oncologia pediatrica. In: Cerlati P Crivelli F, editors. La Musicoterapia in Oncologia e Nelle Cure Palliative. Milano: Franco Angeli (2015). p. 99–115.

[59] Bennett A Sofija E Green B Guerra P Howard F Oliveira A. ‘It’s like a hug’: examining the role of music-making for the well-being of youth during the COVID-19 pandemic. J Youth Stud. (2024):1–17. 10.1080/13676261.2024.2305911

[60] Brannigan P. Queen’s Brian may is now a doctor of science: watch his acceptance speech. (2024). Available online at: https://www.loudersound.com/news/queens-brian-may-is-now-a-doctor-of-science-watch-his-acceptance-speech (accessed December 21, 2024).

[61] Levine N. 4 Artists who ditched their dental careers. (2024). Available online at: https://www.dentalproductsreport.com/view/4-artists-who-ditched-their-dental-careers (accessed December 21, 2024).

[62] Matilda’s Lab. Little answers for little ears. Matilda’s lab hall of fame – Mira Aroyo. (2024). Available online at: https://matildaslab.wordpress.com/2018/03/13/matildas-lab-hall-of-fame-mira-aroyo/ (accessed December 21, 2024).

[63] BBC News. Sir Paul McCartney says artificial intelligence has enabled a ‘final‘ Beatles song. (2024). Available online at: https://www.bbc.com/news/entertainment-arts-65881813 (accessed December 21, 2024).

[64] Jing J. Deep learning-based music quality analysis model. Appl Bionics Biomech. (2022) 2022:6213115. 10.1155/2022/6213115 35733449 PMC9208980

[65] Guo Y Liu Y Zhou T Xu L Zhang Q. An automatic music generation and evaluation method based on transfer learning. PLoS One. (2023) 18(5):e0283103. 10.1371/journal.pone.0283103 37163469 PMC10171593

[66] Pandeya YR Bhattarai B Lee J. Deep-learning-based multimodal emotion classification for music videos. Sensors. (2021) 21(14):4927. 10.3390/s21144927 34300666 PMC8309938

[67] Else H Van Noorden R. The fight against fake-paper factories that churn out sham science. Nature. (2021) 591(7851):516–9. 10.1038/d41586-021-00733-5 33758408

[68] Candal-Pedreira C Ross JS Ruano-Ravina A Egilman DS Fernández E Pérez-Ríos M. Retracted papers originating from paper mills: cross sectional study. Br Med J. (2022) 379:e071517. 10.1136/bmj-2022-071517 36442874 PMC9703783

[69] Christopher J. The raw truth about paper mills. FEBS Lett. (2021) 595(13):1751–7. 10.1002/1873-3468.14143 34180058

[70] Liverpool L. AI Intensifies fight against ‘paper mills’ that churn out fake research. Nature. (2023) 618(7964):222–3. 10.1038/d41586-023-01780-w 37258739

[71] Raglio A Imbriani M Imbriani C Baiardi P Manzoni S Gianotti M Machine learning techniques to predict the effectiveness of music therapy: a randomized controlled trial. Comput Methods Programs Biomed. (2020) 185:105160. 10.1016/j.cmpb.2019.105160 31710983

[72] Modran HA Chamunorwa T Ursuțiu D Samoilă C Hedeșiu H. Using deep learning to recognize therapeutic effects of music based on emotions. Sensors. (2023) 23(2):986. 10.3390/s23020986 36679783 PMC9861051

[73] Wang Z Guan X Li E Dong B. A study on music therapy aimed at psychological trauma recovery for bereaved families driven by artificial intelligence. Front Psychol. (2024) 15:1436324. 10.3389/fpsyg.2024.1436324 39439761 PMC11493750

[74] Music and Science. Sage journals. (2024). Available online at: https://journals.sagepub.com/home/mns (accessed December 21, 2024).

[75] Journal of Music Therapy. Oxford academic. (2024). Available online at: https://academic.oup.com/jmt (accessed December 21, 2024).

[76] Music Therapy Perspectives. Oxford academic. (2024). Available online at: https://academic.oup.com/mtp (accessed December 21, 2024).

[77] Music and Medicine. International association for music & medicine. (2024). Available online at: https://mmd.iammonline.com/index.php/musmed (Last accessed December 21, 2024).

[78] Sear HG. Cure by music. West Lond Med J. (1946) 51:9–15. 20984155

[79] Waugh MO. Music in the post-war therapy. Med Womans J. (1946) 53(4):49. 21022727

[80] Wells S. Music and the mentally ill. Trained Nurse Hosp Rev. (1947) 119(3):186–8. 20261901

[81] Light S. Music and occupational therapy. Can J Occup Ther. (1947) 14(4):76–8. 10.1177/000841744701400403 18910445

[82] Thompson N Bloska J Abington A Masterson A Whitten D Street A. The feasibility and acceptability of neurologic music therapy in subacute neurorehabilitation and effects on patient mood. Brain Sci. (2022) 12(4):497. 10.3390/brainsci12040497 35448028 PMC9029413

[83] Riew GJ Kamal K Hijaz B Awh KC Nambudiri VE. Clinical music interventions and music therapy in dermatology. Arch Dermatol Res. (2023) 315(9):2485–90. 10.1007/s00403-023-02634-1 37208459

[84] Yang S Suh JH Kwon S Chang MC. The effect of neurologic music therapy in patients with cerebral palsy: a systematic narrative review. Front Neurol. (2022) 13:852277. 10.3389/fneur.2022.852277 36176557 PMC9514322

[85] Hill-Wilkes N Renales F Seibenhener S Jefferson LL. Examining the effects of music therapy on decreasing agitation in Alzheimer’s disease. J Holist Nurs. (2023) 42(2):133–42. 10.1177/08980101231198717 37671565

[86] Eseadi C Amedu AN. Potential impact of music interventions in managing diabetic conditions. World J Clin Cases. (2023) 11(13):2916–24. 10.12998/wjcc.v11.i13.2916 37215419 PMC10198074

[87] Pathania S Slater LZ Vose C Navarra AM. Music therapy and pain management in patients with end-stage liver disease: an evidence-based practice quality improvement project. Pain Manag Nurs. (2019) 20(1):10–6. 10.1016/j.pmn.2018.07.004 30448441

[88] Wang J Li W. Improvement effect of PERMA model-based nursing intervention plus music therapy on patients with acute liver failure undergoing plasma exchange therapy. Emerg Med Int. (2022) 2022:2485056. 10.1155/2022/2485056 35811606 PMC9259328

[89] Kobus S Buehne AM Kathemann S Buescher AK Lainka E. Effects of music therapy on vital signs in children with chronic disease. Int J Environ Res Public Health. (2022) 19(11):6544. 10.3390/ijerph19116544 35682129 PMC9180355

[90] Sand-Jecklin K Emerson H. The impact of a live therapeutic music intervention on patients’ experience of pain, anxiety, and muscle tension. Holist Nurs Pract. (2010) 24(1):7–15. 10.1097/HNP.0b013e3181c8e435 20023519

[91] Cao M Zhang Z. Adjuvant music therapy for patients with hypertension: a meta-analysis and systematic review. BMC Complement Med Ther. (2023) 23(1):110. 10.1186/s12906-023-03929-6 37024863 PMC10077636

[92] Adlakha K Mathur MK Datta A Kalsi R Bhandari B. Short-term effect of spiritual music on heart rate variability in medical students: a single-group experimental study. Cureus. (2023) 15(2):e34833. 10.7759/cureus.34833 36919072 PMC10008213

[93] Kulinski J Ofori EK Visotcky A Smith A Sparapani R Fleg JL. Effects of music on the cardiovascular system. Trends Cardiovasc Med. (2022) 32(6):390–8. 10.1016/j.tcm.2021.06.004 34237410 PMC8727633

[94] Lewis A Kal E Nolan CM Cave P Grillo L Conway J Pilot study of physiotherapist-led versus music therapist-led breathing control exercises for young adults living with breathing pattern disorder: a randomised controlled trial protocol. BMJ Open Respir Res. (2022) 9(1):e001414. 10.1136/bmjresp-2022-001414 36104105 PMC9476152

[95] Koelsch S Jäncke L. Music and the heart. Eur Heart J. (2015) 36(44):3043–9. 10.1093/eurheartj/ehv430 26354957

[96] Takmak Ş Karaçar Y Karaçar Hİ Küçükakça Çelik G. The effect of nature-based music intervention on adaptation and anxiety levels in patients with COVID-19 placed in the prone position: a randomized controlled trial. Intensive Crit Care Nurs. (2023) 79:103496. 10.1016/j.iccn.2023.103496 37542800

[97] Santos KVGD Dantas JKDS Fernandes TEL Medeiros KS Sarmento ACA Ribeiro KRB Music to relieve pain and anxiety in cardiac catheterization: a systematic review and meta-analysis. Heliyon. (2024) 10(13):e33815. 10.1016/j.heliyon.2024.e33815 39044980 PMC11263635

[98] de Witte M Spruit A van Hooren S Moonen X Stams GJ. Effects of music interventions on stress-related outcomes: a systematic review and two meta-analyses. Health Psychol Rev. (2020) 14(2):294–324. 10.1080/17437199.2019.1627897 31167611

[99] Chanda ML Levitin DJ. The neurochemistry of music. Trends Cogn Sci. (2013) 17(4):179–93. 10.1016/j.tics.2013.02.007 23541122

[100] Linnemann A Wenzel M Grammes J Kubiak T Nater UM. Music listening and stress in daily life-a matter of timing. Int J Behav Med. (2018) 25(2):223–30. 10.1007/s12529-017-9697-5 29164485 PMC5852177

[101] Sai Nuthalapati B Dey D Singh B Anamika FNU Kanagala SG Garg N Harmonizing hearts: exploring the impact of music therapy on cardiovascular health. Cardiol Rev. (2024). 10.1097/CRD.0000000000000676 38441154

[102] Silletti A Guzzo I Mastrolorenzo A Piga S Atti MCD Grimaldi Capitello T. Effects of live music during hemodialysis treatments in pediatric patients. J Nephrol. (2023) 36(7):2071–9. 10.1007/s40620-023-01717-6 37594670

[103] Sin M Dennis T. Can music therapy and aroma therapy really reduce dental anxiety and fear? Evid Based Dent. (2023) 24(2):59–60. 10.1038/s41432-023-00881-9 37161071

[104] Mata Ferro M Falcó Pegueroles A Fernández Lorenzo R Saz Roy MÁ Rodríguez Forner O Estrada Jurado CM The effect of a live music therapy intervention on critically ill paediatric patients in the intensive care unit: a quasi-experimental pretest-posttest study. Aust Crit Care. (2023) 36(6):967–73. 10.1016/j.aucc.2023.01.006 36868934

[105] Moon JR Song J Huh J Kang IS Kim JH Park SW The effects of music intervention on anxiety and stress responses in adults with CHD undergoing cardiac catheterisation. Cardiol Young. (2023) 33(2):213–20. 10.1017/S1047951122000439 35285439

[106] Vijay A Hauser JM. “This won't hurt a bit”: is there a role for music in bedside procedures? Crit Care Explor. (2023) 5(4):e0900. 10.1097/CCE.0000000000000900 37038394 PMC10082227

[107] Erbay Dalli Ö Bozkurt C Yildirim Y. The effectiveness of music interventions on stress response in intensive care patients: a systematic review and meta-analysis. J Clin Nurs. (2023) 32(11–12):2827–45. 10.1111/jocn.16401 35668626

